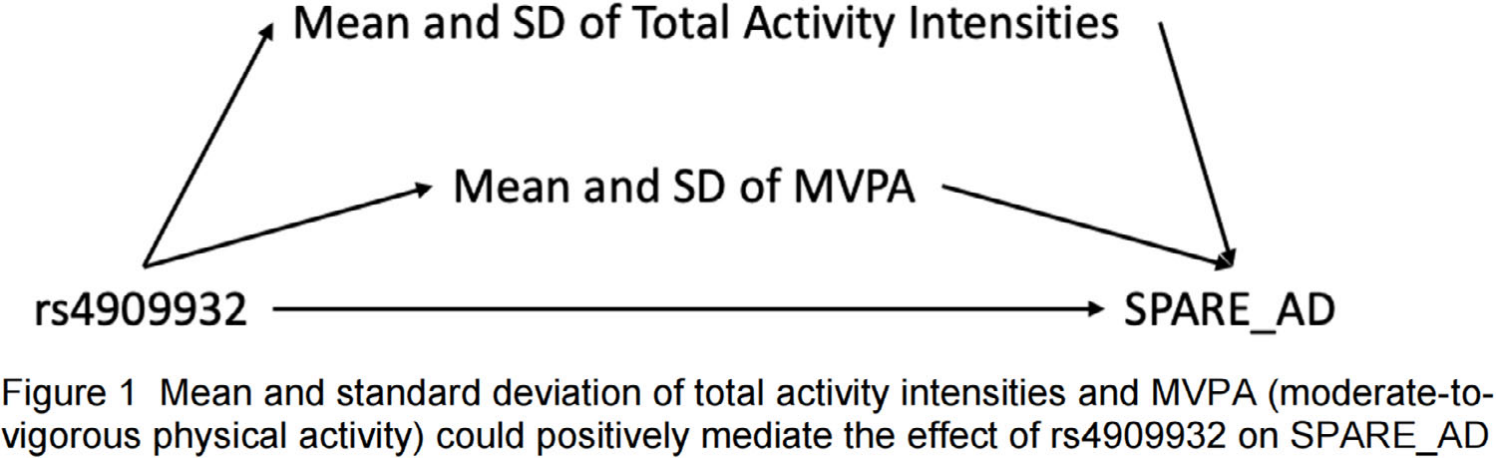# Physical activity pattern mediating the relationship between genetic variants and AD‐like brain atrophy

**DOI:** 10.1002/alz.092392

**Published:** 2025-01-09

**Authors:** Hanxiang Xu, Shizhuo Mu, Jingxuan Bao, Christos Davatzikos, Haochang Shou, Li Shen

**Affiliations:** ^1^ University of Pennsylvania, Philadelphia, PA USA; ^2^ Perelman School of Medicine, University of Pennsylvania, Philadelphia, PA USA

## Abstract

**Background:**

Studies have shown physical activity (PA) patterns are heritable traits and are correlated with several known genetic risk factors including APOE, the best‐known gene associated with Alzheimer’s Disease (AD). SPARE‐AD was a previously developed machine learning index known to be sensitive to AD‐like brain atrophy. However, the relationship between genetic variants, physical activity patterns and AD‐related neuroimaging features have yet been extensively studied due to the lack of appropriate data and statistical methods for handling complex multimodal data. To bridge this gap, we perform a high‐dimensional mediation analysis to decompose the pathway from known genetic variants to AD‐like brain atrophy through accelerometry‐based physical activity patterns.

**Method:**

We obtained genetic, accelerometry and neuroimaging data from UK Biobank (n=13,425). SPARE‐AD index was calculated from structural MRI data. Genetic variants were selected by GWAS using SAIGE with SPARE‐AD index as the outcome, adjusting for years of education, age, gender and the first 10 principal components as covariates. The 7‐day accelerometry data were summarized into conventional statistics such as average total log activity counts (TLAC) and percent of moderate‐to‐vigorous PA (MVPA), and data‐driven features derived using functional Principal Component Analysis. We performed high‐dimensional Bayesian mediation analysis (BAMA) to preliminarily identify the physical activity patterns with non‐zero indirect effect of genetic variants on SPARE‐AD. The mediation effect was later confirmed by significant associations between exposure (genetic variant) and mediator (physical activity pattern) using univariate analysis.

**Result:**

GWAS of SPARE‐AD identified 22 unique SNPs (p<=5e‐8). These SNPs and 154 physical activity features were included in our mediation analyses. We identified 128 significant (SNP, PA, SPARE‐AD) mediation relationships (one example shown in Figure 1). Most of the SNPs are located on AMPD3 gene, which is associated with energy metabolism. Among all the identified mediators, lower TAC and MVPA were identified to be associated with higher SPARE‐AD, and positive indirect effects on the association between SNPs and SPARE‐AD.

**Conclusion:**

We found several significant pathways, where physical activity measures could mediate genetic effects on AD‐related brain atrophy. This could provide insights for developing personalized behavioral intervention strategies that mitigate potential genetic risks.